# Subjective Cognitive Complaints in Newly-Diagnosed Parkinson’s Disease With and Without Mild Cognitive Impairment

**DOI:** 10.3389/fnins.2021.761817

**Published:** 2021-11-24

**Authors:** Chenxi Pan, Jingru Ren, Ping Hua, Lei Yan, Miao Yu, Yajie Wang, Gaiyan Zhou, Ronggui Zhang, Jiu Chen, Weiguo Liu

**Affiliations:** ^1^Department of Neurology, The Affiliated Brain Hospital of Nanjing Medical University, Nanjing, China; ^2^Institute of Brain Functional Imaging, Nanjing Medical University, Nanjing, China; ^3^Institute of Neuropsychiatry, The Affiliated Brain Hospital of Nanjing Medical University, Fourth Clinical College of Nanjing Medical University, Nanjing, China

**Keywords:** Parkinson’s disease, subjective cognitive complaints, mild cognitive impairment, non-motor symptoms, attention/working memory

## Abstract

**Background:** Subjective cognitive complaints (SCCs) and mild cognitive impairment (MCI) are common among patients with Parkinson’s disease (PD). However, the relationship between SCCs and MCI is not well understood. Herein, we aimed to investigate whether there are any differences in the prevalence and risk factors of SCCs between early PD patients with and without MCI.

**Methods:** Overall, 108 newly diagnosed, untreated PD patients underwent comprehensive neuropsychological assessments. PD patients with mild cognitive impairment (PD-MCI) were diagnosed according to the MCI level II criteria. Furthermore, SCCs were measured with the Cognitive Complaints Interview (CCI). Logistic regression analysis, after adjusting for confounding variable, was performed in order to investigate risk factors of SCCs in PD-MCI patients and PD patients with normal cognition (PD-NC).

**Results:** Furthermore, 42 (42.3%) participants reported SCCs and 53 (53.5%) participants were diagnosed with PD-MCI. The prevalence of SCCs in PD-MCI and PD-NC participants was 30.3% and 12.1%, respectively. Logistic regression analyses revealed that the presence of SCCs in PD-MCI group was significantly associated with Non-Motor Symptoms Questionnaire (NMSQ) score (OR = 1.340, 95%CI = 1.115−1.610, *p* = 0.002), while the presence of SCCs in PD-NC group was significantly associated with time of Stroop Color-Word Test card C (OR = 1.050, 95%CI = 1.009−1.119, *p* = 0.016).

**Conclusion:** SCCs are frequent among patients with early PD. The prevalence and risk factor of SCCs are distinct in PD with and without MCI. These findings suggest that SCCs in early PD with different cognitive status appear to have different pathogenicity.

## Introduction

Cognitive impairment is one of the most important non-motor symptoms (NMSs) of Parkinson’s disease (PD; Aarsland et al., 2021), even in the early stages of the disease (Aarsland et al., 2009; Weil et al., 2018). The full spectrum of cognition occurs among individuals with PD, which ranges from normal cognition through mild cognitive impairment (MCI) to PD dementia (PDD; Aarsland et al., 2017). MCI is an intermediate stage that occurs between normal cognition and dementia in PD (Caviness et al., 2007). Approximately 50% of PD patients with normal cognition (PD-NC) develop MCI within 6 years (Pigott et al., 2015). Meanwhile, after 5 years, 27.8% of PD patients with mild cognitive impairment (PD-MCI) patients revert to normal cognition (Pedersen et al., 2017), and almost 40% of PD patients with MCI (PD-MCI) go on to develop PDD (Pedersen et al., 2017). During the past decade, MCI have become increasingly recognized as an important pre-dementia stage of PD.

Subjective cognitive complaints (SCCs) are subjectively identified cognitive decline of people who, may or may not, have detected impairment on neuropsychological tests (Barbosa et al., 2019). A meta-analysis suggests that elderly people with SCCs are at increased risk of MCI and dementia (Mitchell et al., 2014). SCCs are also common among PD patients (Lehrner et al., 2014), with a prevalence that ranges from 30.3 to 60% (Erro et al., 2014; Baschi et al., 2018). Approximately 70% of PD-NC patients who develop cognitive impairment over time have a cognitive complaint at baseline (Purri et al., 2020), and SCCs have been suggested to be an independent predictor of development of MCI in cognitively normal PD patients (Erro et al., 2014; Hong et al., 2014). Furthermore, Hong et al. demonstrated that, in whole PD cohort, SCCs were correlated with neuropsychological tests (Hong et al., 2018). However, among non-demented PD subjects, there was little evidence to suggest that SCCs were related to impaired cognitive testing (AlDakheel et al., 2019), and a longitudinal research showed that depression occured secondary to or independent of cognitive complaints (Purri et al., 2020). The inconsistency of results may be due to the included PD patinets with different cognitive status, for Baschi et al. (2018) suggested SCCs in PD with or without MCI seemed to have a different etiology. Besides, some studies have also discovered that SCCs are correlated with psychiatric symptoms (Lehrner et al., 2014; Chua et al., 2021). Therefore, the relationship of SCCs, MCI and clinical factors (e.g., neuropsychological tests, psychiatric symptoms) is not yet well understood.

In this study, we hypothesized that the prevalence and risk factors of SCCs among early PD patients differ depending on cognitive status. Using a cohort of newly diagnosed, untreated PD patients, this study aims to: (1) investigate the prevalence of SCCs in early PD patients with different cognitive status, and (2) examine the implicated risk factors of SCCs in early PD-MCI and PD-NC patients.

## Materials and Methods

### Participants

All participants with PD were diagnosed by a neurologist specializing in movement disorders. Patients were enrolled in the study *via* the Affiliated Brain Hospital of Nanjing Medical University. The inclusion criteria were: (1) age between 40∼80 years old; (2) be newly diagnosed with idiopathic PD based on the United Kingdom Parkinson’s Disease Society Brain Bank Clinical Diagnostic Criteria (Gibb and Lees, 1988); (3) clinical follow-up continued at least 1 year; (4) were not taking any antiparkinsonian drugs; and (5) were Chinese native speakers. Participants were excluded if: (1) they were diagnosed with atypical or secondary Parkinsonism disorders; (2) had a history of dementia, cancer, diabetes, hyperthyroidism, or severe psychiatric illnesses; (3) history of serious diseases that could influence cerebral function (i.e., severe head injury, cerebrovascular disease, and brain operation); (4) were illiterate; and (5) took antidepressants or anticholinergic drugs. In addition, participants were excluded if they had incomplete neuropsychological testing data, which was defined as missing any test in memory, visuospatial or language domain, as well as more than one test in attention or executive domain.

### Standard Protocol, Approvals, Registrations, and Patient Consents

Approval from the Medical Ethics Committee of the Affiliated Brain Hospital of Nanjing Medical University was obtained prior to study initiation. Written informed consent was obtained from all patients.

### Clinical Assessment

Demographic and clinical information were collected. All PD patients were assessed using the Unified Parkinson’s Disease Rating Scale Part II and part III (UPDRS-II, UPDRS-III). Clinical phenotype was evaluated using the UPDRS tremor dominant (TD), postural instability/gait difficulty (PIGD), and indeterminate classification (Stebbins et al., 2013). Disease stage was determined using the Hoehn and Yahr (H-Y) Scale (Hoehn and Yahr, 1967), NMSs were evaluated using the Non-Motor Symptoms Questionnaire (NMSQ; Chaudhuri et al., 2006). The severity of depression and anxiety were evaluated utilizing the Hamilton Depression Rating Scale (HAMD; Hamilton, 1960) and the Hamilton Anxiety Scale (HAMA; Hamilton, 1959), respectively.

### Cognitive Assessment

Global cognitive function was assessed using the Mini-Mental State Examination (MMSE; Folstein et al., 1975) and Montreal Cognitive Assessment (MoCA; Nasreddine et al., 2005; Lu et al., 2011). Moreover, all patients underwent a formal, comprehensive neuropsychological battery, as administered by trained researchers. As recommended by the Movement Disorder Society (MDS) Task Force (Litvan et al., 2012), a neuropsychological battery include five cognitive domains, which were evaluated with two or more tests. These include: (1) Digit Span Backward Test (DST), Trail Making Test A (TMT-A), and Stroop Color-Word Test (SCWT; comprising three tasks) assessing attention and working memory; (2) Trail Making Test B (TMT-B), Clock Drawing Test (CDT), and Verbal Fluence Test (VFT) measuring executive function; (3) Auditory Verbal Learning Test (AVLT) and Logical Memory Test (LMT) measuring memory; (4) Benton’s Judgment of Line Orientation Test (JLOT) and Hooper Visual Organization Test (HVOT) assessing visuospatial function; (5) Boston Naming Test (BNT) and Wechsler Adult Intelligence Scale III (WAIS-III) Similarities Test measuring language.

PD patients with mild cognitive impairment met MCI Level II diagnostic criteria proposed by the MDS (Litvan et al., 2012): impaired performance, with scores 1.5 standard deviations (SDs) below normative data for score adjustment (based on age, gender and education), on at least two tests of neuropsychological battery. PD patients who did not fulfilled the diagnostic criteria for PD-MCI were classified as PD-NC.

Subjective cognitive complaints were assessed utilizing the Cognitive Complaints Interview (CCI; Catherine et al., 2006), which consisted of 10 questions regarding the changes of cognitive function. The presence of SCCs was defined as CCI score over 3 (Yoo et al., 2020). Then, the PD-MCI patients and PD-NC patients were divided into two groups, including those with subjective cognitive complaints (SCCs^+^) and those without cognitive complaints (SCCs^–^).

### Statistical Analysis

Demographic and clinical data was expressed as frequency (percent) and mean (standard deviation), respectively, for categorical and continuous variables. Student *t*-test and Mann-Whitney U test were used to analyze continuous variables between the two groups (SCCs^+^ and SCCs^–^). A Chi-square test was utilized to analyze categorical variables.

A binary logistic regression analysis was carried out to investigate the independent effect of risk factors for development of SCCs after adjusting for several other factors (e.g., age, sex, education) or adjusting for confounding variable (e.g., depression). Parameters associated with outcome at the univariate analysis (univariate *p* value < 0.10) were included in the regression analysis.

Statistical significance was set at *p* < 0.05. All statistical analyses of data were performed with the Statistical Package for the Social Sciences (SPSS) (version 25).

## Results

### Demographic and Clinical Characteristics

Overall, 108 participants with complete baseline assessments were included in the study. Among the participants, eight participants were excluded and one PD patient were excluded for incomplete neuropsychological assessment data. Finally, 99 newly diagnosed, untreated PD patients were included in the subsequent analyses. Baseline demographic and clinical features are described in Table 1. Among 99 early PD patients, 53 (53.5%) fulfilled the diagnosis of MCI, and 42 (42.3%) reported SCCs. Results indicated that SCCs were more prevalent among patients with MCI compared to those without (30/53 vs 12/46, respectively, Chi-squared test, *P* = 0.002). According to SCCs, the PD-MCI group and PD-NC group were both stratified into two subgroups, including (1) PD patients with MCI and SCCs (PD-MCI + SCC, *n* = 30, 30.3%), (2) PD patients with MCI, but no SCCs (PD-MCI-SCC, *n* = 23, 23.2%), (3) PD patients with SCCs, but no objective cognitive decline (PD-NC + SCC, *n* = 12, 12.1%), and (4) PD patients with neither SCCs nor objective cognitive decline (PD-NC-SCC, *n* = 34, 34.4%). The PD-MCI + SCC group were found to have significantly higher UPDRS-II, NMSQ, HAMA, and HAMD scores than the PD-MCI-SCC group (*P* < 0.05). Furthermore, the two groups did not differ with regards to age, sex, education, disease duration, H-Y stage, UPDRS-III, clinical phenotype, MMSE, and MOCA. The PD-NC + SCC group were found to have significantly higher NMSQ score than the PD-MCI + SCC group (*P* < 0.05). Additionally, we found no significant differences in age, sex, education, disease duration, H-Y stage, UPDRS-II, UPDRS-III, clinical phenotype, HAMA, HAMD, MMSE, and MOCA between PD-NC + SCC group and PD-NC-SCC group.

**TABLE 1:** The clinical and demographic characteristics of cognitively impaired and cognitively normal PD patients with and without subjective cognitive complaints.

	PD-MCI (*n* = 53, 53.5%)		*P* *	PD-NC (*n* = 46, 46.5%)		*P* *
	SCCs^+^ (*n* = 30, 30.3%)	SCCs^–^ (*n* = 23, 23.2%)		SCCs^+^ (*n* = 12, 12.1%)	SCCs^–^ (*n* = 34, 34.4%)	
Age, years^a^	60.90 ± 6.46	60.87 ± 7.29	0.987	57.08 ± 6.01	55.44 ± 7.37	0.492
Sex, male (%)	11 (36.67%)	7 (30.43%)	0.635	9 (75.0%)	19 (55.9%)	0.243
Education, years	9.42 ± 3.20	9.24 ± 3.36	0.845	10.58 ± 1.68	11.02 ± 3.23	0.662
Disease duration, months	16.03 ± 11.36	15.48 ± 10.74	0.857	19.67 ± 12.00	23.47 ± 16.32	0.464
H-Y stage	1.65 ± 0.51	1.63 ± 0.53	0.892	1.67 ± 0.39	1.46 ± 0.43	0.144
UPDRS-II, score	8.23 ± 3.39	5.78 ± 3.40	**0.012**	8.85 ± 4.12	6.38 ± 3.06	0.057
UPDRS-III, score	22.90 ± 10.65	21.65 ± 10.71	0.675	18.85 ± 7.60	17.56 ± 8.84	0.745
Motor phenotype			0.637			0.463
- TD (%)	7 (23.3%)	6 (26.1%)		3 (25.0%)	7 (20.6%)	
- Indeterminate (%)	7 (23.3%)	3 (13.0%)		3 (25.0%)	4 (20.6%)	
- PIGD (%)	16 (53.3%)	14 (60.9%)		6 (50.0%)	23 (67.6%)	
NMSQ	9.70 ± 4.89	5.17 ± 3.45	**0.000**	10.33 ± 5.87	7.12 ± 3.72	**0.033**
HAMA	8.10 ± 3.67	4.91 ± 3.27	**0.002**	9.08 ± 10.10	5.41 ± 4.79	0.102
HAMD	12.97 ± 6.10	7.83 ± 5.98	**0.003**	13.33 ± 11.29	8.00 ± 6.57	0.054
MMSE	27.23 ± 1.85	27.83 ± 2.31	0.304	28.17 ± 0.94	28.21 ± 1.82	0.944
MOCA	20.97 ± 2.88	22.48 ± 3.06	0.074	24.58 ± 3.78	25.03 ± 2.86	0.671

*^a^Categorical and continuous variables were reported as frequency (percent) and mean (standard deviation), respectively.*

*Student t-test and Mann-Whitney U test were used for assessment of continuous variables, Chi-square test was utilized for evaluation of categorical variables. Statistically significant differences (p < 0.05) are shown in bold.

*Parkinson’s disease (PD); PD patient with mild cognitive impairment (PD-MCI); PD patient with normal cognition (PD-NC); subjective cognitive complaints (SCCs^+^); without subjective cognitive complaints (SCCs^–^); Hoehn and Yahr (H-Y); Unified Parkinson’s Disease Rating Scale (UPDRS); Tremor Dominant (TD); Postural Instability-Gait Difficulty (PIGD); Non-Motor Symptoms Questionnaire (NMSQ); Hamilton Anxiety Scale (HAMA); Hamilton Depression Rating Scale (HAMD); Mini Mental State Examination (MMSE); Montreal Cognitive Assessment (MOCA).*

### Neuropsychological Assessments in SCC Subgroups

Between the PD-NC subgroups, compared to the PD-NC-SCC group, the PD-NC + SCC group did not perform significantly worse on global cognition and all cognitive tests, with the exception of the SCWT card B time and card C time (*p* < 0.05, uncorrected). If Bonferroni correction was applied, the results were no longer statistically significant (Table 2). In addition, there were no significant differences in global cognition and cognitive tests between the PD-MCI-SCC group and PD-MCI + SCC group (*p* > 0.05) (Supplementary Table 1).

**TABLE 2:** Cognitive assessments of cognitively normal PD patients with and without subjective cognitive complaints.

	Total PD-NC (*n* = 46)	PD-NC + SCC (*n* = 12, 26.09%)	PD-NC-SCC (*n* = 34, 73.91%)	*p* value*
***Attention/Working memory***				
DST	12.61 ± 2.17	12.08 ± 2.58	12.79 ± 2.01	0.334
DST-forward	7.70 ± 1.19	7.17 ± 1.47	7.88 ± 1.04	**0.073**
DST-backward	4.83 ± 1.18	4.92 ± 1.38	4.79 ± 1.12	0.761
TMT-A (s)	73.11 ± 22.71	71.58 ± 19.48	73.65 ± 24.00	0.790
SCWT-A-time	26.22 ± 6.71	28.17 ± 8.82	25.53 ± 5.80	0.246
SCWT-B-time	36.53 ± 8.65^#^	41.58 ± 11.40	34.70 ± 6.72^#^	**0.016**
SCWT-C-time	69.76 ± 20.21^#^	82.67 ± 28.75	65.06 ± 13.86^#^	**0.008**
SCWT-A-right	49.93 ± 0.25	49.83 ± 0.39	49.97 ± 0.17	0.102
SCWT-B-right	49.58 ± 1.22^#^	49.00 ± 2.09	49.79 ± 0.60^#^	**0.053**
SCWT-C-right	48.29 ± 3.37^#^	47.67 ± 4.91	48.52 ± 2.67^#^	0.461
***Executive***				
TMT-B (s)	148.43 ± 31.17	155.00 ± 29.35	146.12 ± 31.88	0.402
CDT	9.63 ± 1.06	9.33 ± 1.56	9.74 ± 0.83	0.264
VFT	20.26 ± 4.37	18.33 ± 4.16	20.94 ± 4.29	**0.075**
***Memory***				
AVLT-delayed recall	6.28 ± 2.22	6.33 ± 1.37	6.26 ± 2.47	0.928
AVLT-recognition	22.41 ± 1.53	22.67 ± 1.44	22.32 ± 1.57	0.510
LMT-delayed recall	6.51 ± 1.98	5.96 ± 2.68	6.71 ± 1.68	0.267
***Visuospatial function***				
JLOT	25.30 ± 2.32	25.83 ± 2.55	25.12 ± 2.24	0.363
HVOT	17.58 ± 3.59	17.75 ± 4.14	17.52 ± 3.44	0.848
***Language***				
Similarities	18.28 ± 3.93	17.42 ± 4.70	18.59 ± 3.66	0.381
BNT	25.76 ± 2.34	25.42 ± 2.23	25.88 ± 2.40	0.559

*Raw scores of neuropsychological tests were presented as mean ± standard deviation.*

*Student t-test and Mann-Whitney U test were used for assessment of continuous variables. Parameters in the univariate analysis (p < 0.10) are shown in bold.

*^#^One participant did not complete the test.*

*Second (s); Parkinson’s disease with normal cognition (PD-NC); PD-NC with subjective cognitive complaints (PD-NC + SCC); PD-NC without subjective cognitive complaints (PD-NC-SCC); Digit Span Backward Test (DST); Trail Making Test A/B (TMT-A/B); Stroop Color-Word Test (SCWT); Clock Drawing Test (CDT); Verbal Fluence Test (VFT); Auditory Verbal Learning Test (AVLT); Logical Memory Test (LMT); Judgment of Line Orientation Test (JLOT); Hooper Visual Organization Test (HVOT); Boston Naming Test (BNT).*

### Regression Analysis

Binary logistic regression analysis revealed that NMSQ score was significantly associated with presence of SCCs in PD-MCI patients, after adjusting for sex, age, education, and depression (OR = 1.340, 95%CI = 1.115−1.610, *p* = 0.002). However, in PD-NC patients, the time of SCWT card C was significantly associated with presence of SCCs after adjusting for sex, age, education, and depression (OR = 1.050, 95%CI = 1.009−1.119, *p* = 0.016) (Table 3).

**TABLE 3:** Multivariate analysis of the PD-MCI group and PD-NC group.

	β	Standard Error of β	AdjORs (95% CI)	*p* value
**PD-MCI**				
NMS	0.293	0.094	1.340 (1.115−1.610)	**0.002**
**PD-NC**				
SCWT-B-right	−0.680	0.404	0.507 (0.230−1.119)	0.093
SCWT-C-time	0.049	0.020	1.050 (1.009−1.119)	**0.016**

*Parkinson’s disease with mild cognitive impairment (PD-MCI); Parkinson’s disease with normal cognition (PD-NC); Odds Ratios (OR); 95% confidence intervals (95% CI). AdjORs: ORs have been adjusted by sex, age, education, and depression. Statistically significant differences (p < 0.05) are shown in bold.*

## Discussion

In the present study, we investigated the frequency of SCCs and its relationship to clinical factors, including neuropsychological tests and NMSs, among early PD patients with different cognitive status. The results of present study demonstrate that more than half of PD-MCI patients reported SCCs, while less than a third of PD-NC patients showed SCCs. In addition, although they don’t fit the diagnostic criteria for MCI, cognitively normal PD patients with SCCs performed worse in some cognitive measures evaluating attention/working memory, compared with those without SCCs. Finally, the PD-NC patients who did worse on SCWT were more likely to report experiencing SCCs. However, the PD-MCI patients who had more NMSs were more likely to report SCCs. Our findings suggest that SCCs in early PD patients have different implications that are dependent on the underlying cognitive status.

Herein, we discovered that 42.3% of newly diagnosed non-demented PD patients report experiencing SCCs, which is higher than previous reports (Lehrner et al., 2014; Chua et al., 2021). The reasons for this discrepancy may be due to a more appropriate subjective measure. The CCI includes 10 questions which explore the cognitive, memory and behavioral domains (Catherine et al., 2006). Thus, it seems to be more appropriate than single yes/no question (i.e., “Do you feel that your memory or thinking have gotten worse?”) to assess SCCs in PD. Indeed, among the few studies that have evaluated the prevalence of SCCs among newly diagnosed PD patients with MCI. Erro et al. (2014) have discovered that 30.3% of 76 newly diagnosed, untreated PD complained of subjective memory impairment (SMI), and approximately 43.4% fulfilled MDS recommend level I MCI criteria. However, this study did not use a standardized instrument to assess cognitive complaint, only relying on a single question in the NMSQ regarding the act of forgetting. Additionally, the author did not evaluate the prevalence of SCC in PD isolation or within the MCI frame. The prevalence of PD-NC + SCC in our cohort was 12.1%, while PD-MCI + SCC accounted for 30.3%. These data are slightly different from Baschi’s research, which demonstrated that 40 (27.2%) PD-NC patients and 48(32.6%) PD-MCI patients reported SCCs (Baschi et al., 2018). Yet, it’s worth noting that the PD patients that the authors concerned about are in mid-moderate stage of the disease, and may be more prone to significant cognitive decline. On the contrary, the prevalence of PD-MCI + SCC in Early Parkinson’s Disease Longitudinal Singapore (PALS) study was 10.7% (Chua et al., 2021), which is lower than our results. This discrepancy is likely due to a simple subjective measure, which may not be the most comprehensive measure for SCC (Kjeldsen and Damholdt, 2019). Besides, the PD-MCI patients in the PALS study are older than those of our research, and older patients are more likely to be aware that their cognitive difficulties are associated with general ageing, instead of PD, and, as a result, do not complain. Hence, differences between present and previous study results might rely on patient clinical characteristics (i.e., age and stage of the disease), diverse assessment measures, and different diagnostic criteria for MCI and SCCs.

Regarding performance on neuropsychological tests, data in our research revealed that the PD-NC + SCC group performed significantly worse with regards to attention/working memory than PD-NC-SCC, while the PD-MCI + SCC group did substantially at the same level of PD-MCI-SCC across all neuropsychological tests. In newly diagnosed PD patients, attention/working memory was the most frequently affected domain (Monastero et al., 2018). In addition, a previous study has that found that cognitive changes in attention/working memory were present in prodromal PD (Darweesh et al., 2017; Weintraub et al., 2017). Thus, our results suggest the presence of cognitive complaints in PD-NC patients may represent a stage of cognitive decline between normal cognition and MCI, as has been reported in the general population (Reid and Maclullich, 2006; Reisberg and Gauthier, 2008; Jessen et al., 2010). Additionally, SCC in preclinical Alzheimer’s disease (AD) is related to increased risk of conversion to dementia (Jessen et al., 2014; Mitchell et al., 2014). The presence of SCCs in PD is an independent risk factor for MCI and, at the same time, can be used as a predictor of future cognitive decline (Hong et al., 2014). Consequently, the report of SCCs, simple to perform, is meaningful in clinical work, which may alert clinicians to focus on early attention/working memory changes. Then, the clinicians know when to start comprehensive neuropsychological assessment evaluation and establish a clear baseline.

Interestingly, further studies have demonstrated that SCCs in PD-MCI and PD-NC groups are associated with different risk factors. We discovered that the former is independently associated with a score of NMSQ, whereas the latter relates to SCWT. Previous studies on the relationships between SCCs and clinical features (i.e., objective cognition test), behavioral symptoms (i.e., depression, apathy, and anxiety) in PD led to conflicting results (Erro et al., 2014; Lehrner et al., 2014; Hong et al., 2018; AlDakheel et al., 2019). A previous report has proposed that subthreshold depression is associated with SCCs in non-demented PD (Santangelo et al., 2014). Lehrner et al. (2014) revealed that depression and cognitive state is significantly associated with SCCs in PD, but Chua et al. (2021) found that in PD-MCI patients, their mood symptoms were not associated with SCCs. Notably, a recent research from the Parkinson’s Progression Marker Initiative (PPMI) discovered that minor hallucinations were associated with subjective cognitive decline (Bejr-Kasem et al., 2021). In addition, there is literature to suggest that, with the exception of various psychiatric disorders, personality traits, such as anxiety and sensitivity, are related to subjective cognitive decline (Jessen et al., 2014). Therefore, SCCs cannot be explained by separate NMS, which is a significant feature of early PD stage (O’Sullivan et al., 2008). NMSQ is developed to further understand the basis and clinical burden of NMS in PD (Breen and Drutyte, 2012). In addition, it can be divided into nine domains that range from sleep dysfunction, urinary, mood, and sexual to cognitive problems (Chaudhuri et al., 2007). Higher NMSQ scores are known to strongly influence quality of life (QoL; Zipprich et al., 2021), which may remind patients of their cognitive impairment. Consequently, our result that the number of NMS is related to SCCs in PD-MCI may be more robust. On the other hand, in line with previous studies (Yoo et al., 2020), the PD-NC + SCC group did worse in cognition than in the PD-NC-SCC group, especially in SCWT, which reflects attention, working memory, executive function, processing speed and cognitive flexibility (Scarpina and Tagini, 2017), despite the fact that both of their cognition performance are above appropriate norms. Furthermore, a neuroimaging study demonstrated that PD patients with SCCs had poorer performance on tasks related to attention, and more severe cortical atrophy in the anterior cingulate gyrus and right inferior parietal lobule, compared to those without (Hong et al., 2012). Ultimately, These results illustrate that SCCs in PD-NC participants may represent an early sign of the potential pathology of PD.

There exist a few limitations in our study. Firstly, CCI has not been validated in PD, and has been answered only by the patients. But Hong et al. (2018) found that CCI was strongly correlated with objective cognitive function in PD, and was used in many researches of subjective cognitive function (Yoo et al., 2020). What’s more, at present, there are neither authoritative recommendations for suitable assessment tool of SCCs, nor any available guidelines recommended someone that should be regarded as the most reliable when obtaining information from PD patients, caregivers and clinicians (Kjeldsen and Damholdt, 2019). Secondly, all two-by-two comparisons in our research were made without correction for multiple comparison. For our study was exploratory, the purpose of two-by-two comparison was to explore the association between SCCs presence and clinical factors (e.g., neuropsychological tests, and psychiatric symptoms) in PD-MCI and PD-NC groups. Therefore, we did not perform multiple comparison analysis test so as to minimize possible Type II errors resulting from stringent correction. Thirdly, it was a cross-sectional study that merely demonstrates association and the sample was relatively small. Yet, this study is a part of longitudinal study. We’ve been recruiting volunteers to participate, and the follow-up of patients has been proceeding in order. Further studies with larger samples, and follow-up data would be able to confirm these findings. At last, the paticipants of study were recruited in hospital, and a possible selection bias cannot be excluded.

## Conclusion

In conclusion, we have demonstrated that SCC is frequently seen in early PD subjects with or without MCI. Furthermore, we found that the attention/working memory of cognitively normal PD patients with SCCs declined. Our findings can help clinicians identify patients who are at risk for cognitive decline in the future, and provide a new understanding of possible neuropathology in PD cognitive dysfunction.

## Data Availability Statement

The original contributions presented in the study are included in the article/Supplementary Material, further inquiries can be directed to the corresponding author/s.

## Ethics Statement

The studies involving human participants were reviewed and approved by the Medical Ethics Committee of The Affiliated Brain Hospital of Nanjing Medical University. The patients/participants provided their written informed consent to participate in this study.

## Author Contributions

WL, JC, and CP made substantial contributions to the conception or design of the work. CP, PH, LY, YW, GZ, and RZ made substantial contributions to the acquisition of the data. CP, JR, and MY made substantial contributions to the analysis of the data. CP, JC, and WL made substantial contributions to the interpretation of the data. CP drafted the work. All authors contributed to the article and approved the submitted version.

## Conflict of Interest

The authors declare that the research was conducted in the absence of any commercial or financial relationships that could be construed as a potential conflict of interest.

## Publisher’s Note

All claims expressed in this article are solely those of the authors and do not necessarily represent those of their affiliated organizations, or those of the publisher, the editors and the reviewers. Any product that may be evaluated in this article, or claim that may be made by its manufacturer, is not guaranteed or endorsed by the publisher.
