# Supplementary material for: Subjective Cognitive Complaints in Newly-Diagnosed Parkinson’s Disease With and Without Mild Cognitive Impairment
Source: Front Neurosci. 2021 Nov 24;15:761817. doi: 10.3389/fnins.2021.761817 (PMC8651703; doi:10.3389/fnins.2021.761817)
Supplement: Supplementary file 1 [file Table_1.docx]

Supplementary Table 1. Cognitive assessments of mild cognitive impaired PD patients with and without subjective cognitive complaints

|  | Total PD-MCI  (n = 53） | PD-MCI+SCC^+^  (n = 30, 56.6%) | PD-MCI-SCC  (n = 23, 43.4%) | P* |  |
| --- | --- | --- | --- | --- | --- |
| ***Attention/Working memory*** | |  |  |  |  |
| DST  DST-forward  DST-backward  TMT-A (s)  SCWT-A-time  SCWT-B-time  SCWT-C-time  SCWT-A-right  SCWT-B-right  SCWT-C-right | 11.13 ± 2.40  6.96 ± 1.59  4.17 ± 1.27  117.67 ± 51.71  30.96 ± 9.68^#^  45.87 ± 18.07^#^  80.29 ± 28.70^#^  49.88 ± 0.43^#^  48.58 ± 2.83^#^  46.79 ± 4.77^#^ | 10.73 ± 2.29  6.80 ± 1.61  3.93 ± 1.08  120.14 ± 43.89  31.67 ± 8.49  45.17 ± 14.04  79.03 ± 27.60  49.80 ± 0.55  48.23 ± 3.45  46.20 ± 5.28 | 11.65 ± 2.50  7.17 ± 1.59  4.48 ± 1.44  114.57 ± 61.05  30.00 ± 11.24^#^  46.82 ± 22.78^#^  82.00 ± 30.71^#^  50.00 ± 0.00^#^  49.05 ± 1.59^#^  47.59 ± 3.95^#^ | 0.170  0.402  0.122  0.704  0.545  0.748  0.717  0.096  0.310  0.303 |  |
| ***Executive***  TMT-B (s)  CDT  VFT | 238.88 ± 107.89  8.08 ± 2.48  16.40 ± 5.59 | 234.79 ± 88.18  8.20 ± 2.47  16.80 ± 7.10 | 244.04 ± 130.52  7.91 ± 2.54  15.87 ± 2.62 | 0.762  0.680  0.553 |  |
| ***Memory***  AVLT-delayed recall  AVLT-recognition  LMT-delayed recall | 4.36 ± 2.67  21.02 ± 2.78  4.63 ± 2.51 | 4.17 ± 2.53  20.50 ± 3.01  4.28 ± 2.44 | 4.61 ± 2.64  21.70 ± 2.34  5.09 ± 2.57 | 0.539  0.122  0.251 |  |
| ***Visuospatial function*** | |  |  |  |  |
| JLOT  HVOT | 22.69 ± 3.36  12.17 ± 4.26 | 22.92 ± 2.61  12.13 ± 4.32 | 22.39 ± 4.18  12.22 ± 4.29 | 0.577  0.944 |  |
| ***Language***  similarities  BNT | 13.91 ± 4.35  22.17 ± 4.00 | 13.47 ± 4.30  22.27 ± 3.16 | 14.48 ± 4.45  22.04 ± 4.96 | 0.407  0.843 |  |

Data are presented as mean ± standard deviation. * Student t test and Mann-Whitney U test for continuous variables. ^#^ One participant did not complete the test. Abbreviations: second(s); Parkinson’s disease with mild cognitive impairment (PD-MCI); PD-MCI with subjective cognitive complaints (PD-MCI+SCC); PD-MCI without subjective cognitive complaints (PD-MCI-SCC); Digit Span Backward Test (DST); Trail Making Test A/B (TMT-A/B); Stroop Color-Word Test (SCWT); Clock Drawing Test (CDT); Verbal Fluence Test (VFT); Auditory Verbal Learning Test (AVLT); Logical Memory Test (LMT); Judgment of Line Orientation Test (JLOT); Hooper Visual Organization Test (HVOT); Boston Naming Test (BNT).
